# Supplementary material for: MetaQTL: a package of new computational methods for the meta-analysis of QTL mapping experiments
Source: BMC Bioinformatics. 2007 Feb 8;8:49. doi: 10.1186/1471-2105-8-49 (PMC1808479; doi:10.1186/1471-2105-8-49)
Supplement: Additional File 7 — MetaQTL Package : jar file and tutorial. This Zip archive contains both the MetaQTL JAR file and the files of the tutorial. [file 1471-2105-8-49-S7.zip › org.inra.metaqtl/doc/org/thalia/bio/entity/LocusContainer.html]

LocusContainer


|  |  |  |
| --- | --- | --- |
| **Overview** \| **Package** \| **Class** \| **Use** \| **Tree** \| **Deprecated** \| **Index** \| **Help** | |  |
| **PREV CLASS**   **NEXT CLASS** | **FRAMES**    **NO FRAMES**     **All Classes** |  |
| SUMMARY: NESTED \| FIELD \| CONSTR \| METHOD | DETAIL: FIELD \| CONSTR \| METHOD |  |


---


## org.thalia.bio.entity Class LocusContainer

```
java.lang.Object
  org.thalia.bio.entity.BioEntity
      org.thalia.bio.entity.BioEntityContainer
          org.thalia.bio.entity.LocusContainer
```

**All Implemented Interfaces:**: IBioAdaptable, IBioEntity, IBioLGroup

**Direct Known Subclasses:**: LGroup

---

``` public abstract class LocusContainer extends BioEntityContainer implements IBioLGroup ```

Class Description Here

**Author:**
:   Jean-Baptiste Veyrieras

---

**Field Summary**

**Fields inherited from class org.thalia.bio.entity.BioEntityContainer**

`entities`

**Fields inherited from class org.thalia.bio.entity.BioEntity**

`name, parent, properties`


**Constructor Summary**

`LocusContainer()`

`LocusContainer(java.lang.String name,
IBioEntity parent)`


| **Method Summary** | |
| --- | --- |
| `void` | `addLocus(IBioLocus locus)` |
| `IBioGenome` | `getGenome()` |
| `IBioLocus` | `getLocus(java.lang.String name)` |
| `int` | `getLocusNumber()` |
| `abstract  int` | `getType()`             There are 2 main class of entities. |
| `IBioLocus[]` | `loci()` |
| `void` | `removeLocus(java.lang.String name)` |
| `void` | `setGenome(IBioGenome genome)` |
| `void` | `setLoci(IBioLocus[] loci)` |

**Methods inherited from class org.thalia.bio.entity.BioEntityContainer**

`addEntity, entities, entityNumber, getEntity, removeEntity`

**Methods inherited from class org.thalia.bio.entity.BioEntity**

`getName, getParent, getProperties, newBioEntity, setName, setProperties`

**Methods inherited from class java.lang.Object**

`clone, equals, finalize, getClass, hashCode, notify, notifyAll, toString, wait, wait, wait`

**Methods inherited from interface org.thalia.bio.IBioEntity**

`getName, getParent, getProperties, setName, setProperties`

**Methods inherited from interface org.thalia.bio.IBioAdaptable**

`getBioAdapter`

**Constructor Detail**

### LocusContainer

```
public LocusContainer()
```

---


### LocusContainer

```
public LocusContainer(java.lang.String name,
                      IBioEntity parent)
```

**Parameters:**: `name` -: `parent` -


**Method Detail**

### getType

```
public abstract int getType()
```

:   **Description copied from interface: `IBioEntity`**
:   There are 2 main class of entities. The first one deals with population
    biological entity, i.e population itself and individuals. The second class
    is a representation of microscopic biological entity from genome container
    to alleles.

    :   **Specified by:**: `getType` in interface `IBioEntity` **Specified by:**: `getType` in class `BioEntity`

---


### getGenome

```
public IBioGenome getGenome()
```

:   **Specified by:**: `getGenome` in interface `IBioLGroup`

---


### setGenome

```
public void setGenome(IBioGenome genome)
```

:   **Specified by:**: `setGenome` in interface `IBioLGroup`

---


### loci

```
public IBioLocus[] loci()
```

:   **Specified by:**: `loci` in interface `IBioLGroup`

---


### addLocus

```
public void addLocus(IBioLocus locus)
```

:   **Specified by:**: `addLocus` in interface `IBioLGroup`

---


### getLocus

```
public IBioLocus getLocus(java.lang.String name)
```

:   **Specified by:**: `getLocus` in interface `IBioLGroup`

---


### removeLocus

```
public void removeLocus(java.lang.String name)
```

:   **Specified by:**: `removeLocus` in interface `IBioLGroup`

---


### getLocusNumber

```
public int getLocusNumber()
```

:   **Specified by:**: `getLocusNumber` in interface `IBioLGroup`

---


### setLoci

```
public void setLoci(IBioLocus[] loci)
```

:   **Specified by:**: `setLoci` in interface `IBioLGroup`


---


|  |  |  |
| --- | --- | --- |
| **Overview** \| **Package** \| **Class** \| **Use** \| **Tree** \| **Deprecated** \| **Index** \| **Help** | |  |
| **PREV CLASS**   **NEXT CLASS** | **FRAMES**    **NO FRAMES**     **All Classes** |  |
| SUMMARY: NESTED \| FIELD \| CONSTR \| METHOD | DETAIL: FIELD \| CONSTR \| METHOD |  |


---
